# Application of Causal Forest Model to Examine Treatment Effect Heterogeneity in Substance Use Disorder Psychosocial Treatments

**DOI:** 10.1002/mpr.70011

**Published:** 2024-12-27

**Authors:** Ryoko Susukida, Masoumeh Amin‐Esmaeili, Elena Badillo‐Goicoechea, Trang Q. Nguyen, Elizabeth A. Stuart, Michael Rosenblum, Kelly E. Dunn, Ramin Mojtabai

**Affiliations:** ^1^ Department of Mental Health Johns Hopkins Bloomberg School of Public Health Baltimore Maryland USA; ^2^ Department of Biostatistics Johns Hopkins Bloomberg School of Public Health Baltimore Maryland USA; ^3^ Department of Health Policy and Management Johns Hopkins Bloomberg School of Public Health Baltimore Maryland USA; ^4^ Department of Psychiatry and Behavioral Sciences Johns Hopkins University School of Medicine Baltimore Maryland USA; ^5^ Department of Psychiatry and Behavioral Sciences Tulane University New Orleans Louisiana USA

**Keywords:** causal forest, heterogeneity, psychosocial interventions, substance use disorder treatment, treatment effect

## Abstract

**Objectives:**

Heterogeneity of treatment effect (HTE) is a concern in substance use disorder (SUD) treatments but has not been rigorously examined. This exploratory study applied a causal forest approach to examine HTE in psychosocial SUD treatments, considering multiple covariates simultaneously.

**Methods:**

Data from 12 randomized controlled trials of nine psychosocial treatments were obtained from the National Institute on Drug Abuse Clinical Trials Network. Using causal forests, we estimated the conditional average treatment effect (CATE) on drug abstinence. To assess HTE, we compared CATE variance against total outcome variability, conducted an omnibus test, and applied the Rank‐Weighted Average Treatment Effect (RATE).

**Results:**

Across nine interventions, CATE variance was lower than total outcome variability, indicating lack of strong evidence of HTE with respect to the baseline covariates considered. The omnibus test and RATE analysis generally support this finding. However, the RATE analysis identified potential HTE in a motivational interviewing trial; this could be a false positive given the multiple analyses; replication is needed to confirm this.

**Conclusions:**

While causal forests show utility in exploring HTE in SUD interventions, limited baseline assessments in most trials suggest a cautious interpretation. The RATE findings for motivational interviewing highlight potential subgroup‐specific treatment benefits, warranting further research.

## 1 Introduction

Heterogeneity of treatment effect (HTE) is a consequence of diverse sub‐phenotypes among persons with SUDs with regard to symptom profile, disease course, recovery trajectory, and singularity of patient's context and a concern for the research and practice of substance use disorder (SUD) treatments (Carroll et al. 2006, 2009; Ball et al. 2007; Winhusen et al. 2008; Weiss et al. 2011; Trivedi et al. 2017; Anderson et al. 2009). Evidence continually reveals that not all SUD treatments are equally efficacious for all patients, but the subgroups who benefit most or least have been difficult to identify (Norcross and Wampold 2011; Grossbard et al. 2016; Hendriks, van der Schee, and Blanken 2012; Riper et al. 2018; Fernandez et al. 2019; Vederhus et al. 2014; Paz Castro et al. 2017; Arroyo, Miller, and Tonigan 2003; Peavy et al. 2017; Haug et al. 2010). Empirically identifying the patient‐level characteristics that are associated with differential effectiveness of SUD treatments could potentially improve treatment outcomes in practice.

Treatment effects may vary across patients with different characteristics. Yet, SUD‐related randomized controlled trials (RCTs) tend to focus on estimating and reporting only the average treatment effect (ATE), which represents the overall treatment effect for all patients. This may mask treatment effects that differ according to patient baseline characteristics, such as differences in drug use history and life circumstances. While crucial to report and consider the ATE, further methods and statistical considerations are needed to identify optimal treatments for different patient groups—the main goal of precision medicine (Hey et al. 2020; Wensink et al. 2021).

Currently, subgroup analysis (i.e., estimating impacts separately within subsamples that are defined by a single covariate, such as race or sex/gender) is the most common method for assessing HTE in SUD research. However, subgroup analysis often involves comparing the estimated treatment effect across various covariates one at a time, without appropriately correcting for multiple testing, which can potentially lead to an increased risk of type I errors (Senn 2004a, 2004b; Tanniou et al. 2016). Additionally, subgroup analysis is prone to elevated type II error as a result of insufficient statistical power to detect effects within subgroups (Senn 2004a, 2004b; Tanniou et al. 2016). General recommendations to deal with these issues are to keep the number of subgroups tested small and to pre‐specify subgroups of interest based on observed effects in the subgroup in previous studies or based on a strong biological/clinical reasoning (Tanniou et al. 2016). However, it is often challenging for researchers to commit in advance to pre‐specified subgroups, and “pre‐analysis plans” may overlook clinically meaningful subgroups with HTE (Athey and Imbens 2016).

A non‐parametric causal forest method developed by Wager and Athey (2018) builds on the widely used random forest algorithm (Breiman 2001) and allows researchers to estimate HTE in the context of many covariates and complex interactions between them. The causal forest approach estimates conditional average treatment effects, that is, the expected treatment effects given patterns of values for a set of covariates; an example pattern may be high school educated women aged 34 who has a history of injection drug use for 6.5 years. The causal forest approach has substantial promise as a method for analyzing HTE (Wager and Athey 2018; Jawadekar et al. 2023; Athey and Wager 2019); however, this approach has not been widely applied to actual RCT data to systematically examine HTE in various clinical domains or in SUD treatment research.

This study was, to our knowledge, the first application of causal forest approach to the SUD field for the purpose of examining HTE within existing SUD treatment RCTs. We applied the causal forest approach to data from 12 National Institute on Drug Abuse (NIDA) Clinical Trial Network (CTN) RCTs that evaluated 9 distinct psychosocial SUD treatments against a treatment‐as‐usual (TAU) condition. We first estimated the conditional average treatment effect (CATE) on drug abstinence given each individual's values on a set of pre‐treatment covariates. Next, we conducted a CATE best linear projection analysis to provide a linear approximation of non‐linear relationship between each pre‐treatment covariate and CATE. We hypothesized that causal forest approach may reveal varying patterns in the CATE, potentially linked to specific covariates, suggesting a degree of variability in treatment responses across different subgroups.

## 2 Methods

### 2.1 Data Sources

RCT data of psychosocial SUD treatments were drawn from the NIDA CTN Data Share Website (National Institute on Drug Abuse) The NIDA CTN studies are nationwide multisite clinical trial studies that evaluate the efficacy of various SUD treatments. In this study, data from 12 RCTs of psychosocial SUD treatments (CTN0004, CTN0005, CTN0006, CTN0007, CTN0013, CTN0014, CTN0015, CTN0021, CTN0031, CTN0037, CTN0044, and CTN0047) were used to examine HTE with a causal forest approach. This study included a total of 5760 participants randomized to 9 distinct psychosocial intervention arms (Motivational Incentives, Motivational Enhancement Therapy, Screening Motivational Assessment, Therapeutic Education System, Brief Strategic Family Therapy, Twelve‐Step Facilitation, Motivational Interviewing, Seeking Safety, and Exercise Program) or a treatment‐as‐usual arm (Table 1). In this study, we pooled data only from trials where the treatment arms were identical. For example, CTN0006 and CTN0007 both compared motivational incentives with treatment as usual, while CTN0004, CTN0013, and CTN0021 examined motivational enhancement therapy, also compared with treatment as usual. Although there was variability in baseline characteristics across trials, the causal forest methodology is designed to account for this variability by estimating the expected treatment effect for each individual based on their baseline covariates. We ensured that there was homogeneity in study design, specifically in terms of treatment arms and consistent measurement of baseline covariates across the pooled trials. This approach allowed us to focus on individual‐level heterogeneity in treatment effects, rather than requiring homogeneity in participant characteristics across trials.

**TABLE 1:** List of the RCTs included in the study.

NIDA study code	Arms	Target drug(s)	*n*
CTN0004 (Ball et al. 2007)	MET versus TAU	Any drugs	461
CTN0013 (Winhusen et al. 2008)	MET versus TAU	Any drugs	200
CTN0021 (Carroll et al. 2009)	MET versus TAU	Any drugs	436
CTN0006 (Killeen et al. 2007)	MInc versus TAU	Stimulant	454
CTN0007 (Stitzer et al. 2007)	MInc versus TAU	Stimulant	388
CTN0047 (Bogenschutz et al. 2014)	SBIRT or SAR versus MSO	Any drugs^a^	1285
CTN0044 (Campbell et al. 2014)	TES versus TAU	Any drugs	507
CTN0014 (Robbins et al. 2011)	BSFT versus TAU	Any drugs	480
CTN0031 (Donovan et al. 2013)	TSF versus TAU	Stimulant	471
CTN0005 (Carroll et al. 2006)	MInt versus TAU	Any drugs	423
CTN0015 (Hien et al. 2009)	SS versus HE	Any drugs	353
CTN0037 (Sanchez et al. 2017)	VIHDE versus HE	Stimulant	302

Abbreviations: BSFT, brief strategic family therapy; HE, health education; MET, motivational enhancement treatment; MInc, motivational incentive; MInt, motivational interviewing; MSO, minimal screening only; SAR, screening assessment and referral; SBIRT, screening brief intervention and referral to treatment; SS, seeking safety; TAU, treatment as usual; TES, therapeutic education system; TSF, twelve‐step facilitation intervention; VIHDE, vigorous intensity high dose exercise.

^a^ Any drugs except for alcohol or nicotine.

### 2.2 Measures

The SUD outcome of interest was abstinence from target substance use at the end of the active trial as determined by biologic testing. Abstinence was determined based on a single negative urine toxicology test result at the end of trials that did not conduct weekly toxicology tests (CTN0005, CTN0014, CTN0031, CTN0047). For studies where a urine toxicology test was conducted weekly during the active phase of trial (CTN0004, CTN0013, CTN0015, and CTN0021), two consecutive negative toxicology test results for the target substance(s) at the end of trial were considered abstinent. For studies where urine samples were collected two or three times a week (CTN0006, CTN0007, CTN0037, and CTN0044), abstinence was defined by the presence of at least one negative toxicology test without any positive tests during the final two weeks of active treatment phase. For CTN00047, where a hair sample was collected instead of urine, abstinence was determined based on a single negative hair toxicology test result at the first follow‐up.

Potential moderators were selected from pre‐treatment covariates suggested in the SUD treatment literature (Norcross and Wampold 2011; Grossbard et al. 2016; Hendriks, van der Schee, and Blanken 2012; Riper et al. 2018; Fernandez et al. 2019; Vederhus et al. 2014; Paz Castro et al. 2017; Arroyo, Miller, and Tonigan 2003; Peavy et al. 2017; Haug et al. 2010). Demographic variables (i.e., age, sex/gender, race, and ethnicity) and socioeconomic variables (i.e., employment status, marital status, years of education, and living arrangement) were available in all 12 RCTs. Most of the baseline clinical and psychosocial variables were derived from the Addiction Severity Index (ASI). The ASI is a semi‐structured interview to assess seven problem areas among individuals with SUDs: medical, employment and support, drug use, alcohol use, legal status, family/social status, and psychiatric status (A. T. McLellan et al. 1992). Composite scores from each problem area were calculated and used as baseline covariates (P. McLellan et al. 1986). In addition to the ASI composite scores, the following variables—which were not part of composite score calculation, were used as baseline covariates: religious preference, medical/psychiatric disability pension status, previous treatments for alcohol/drug use, lifetime physical/sexual abuse history, and injection drug use. ASI was available for 10 RCTs except for CTN0044 and CTN0047, for which we ran a causal forest model with demographic and socioeconomic variables only.

### 2.3 Statistical Analysis

A causal forest is the aggregation of many causal trees that are split into random subsamples of the data. Specifically, each tree partitions the covariate space by recursively splitting multiple times into branches and then ultimately into leaves, where each split is made on one covariate (e.g., on sex/gender, separating males from females, or on age separating those younger than 57 from those of 57 years old and older) to maximize the difference between the treatment effects on the two sides of the split. Based on each tree, there is a noisy estimated treatment effect for each unit in the dataset (based on which leaf of the tree the unit falls into). Such estimates are averaged over all the trees to obtain the forest‐estimated Conditional Average Treatment Effect (CATE).

In our analysis, we implemented causal forests using the “grf” package (Tibshirani et al. 2024). This causal forest implementation first constructs regression forests for the outcome given baseline covariates and for propensity scores. These forests are used to generate kernel weights for robust estimation of the CATE. Full details are in Athey and Wager (2019, Section 1.3).

The CATE on drug abstinence conditional on individuals' covariate values estimated by the causal forest methodology (Wager and Athey 2018) is defined as:

$$\tau \left(x\right)=E\left[{Y_{i}}^{\left(1\right)}-{Y_{i}}^{\left(0\right)}|X_{i}=x_{i}\right],$$

where ${Y_{i}}^{\left(1\right)}$ and ${Y_{i}}^{\left(0\right)}$ respectively represent the outcome value that a random individual from the population would have experienced under active treatment and under treatment as usual (the control condition), and x is a specific pattern of values of these covariates. A tree b estimates the treatment effect by the difference between average outcomes between the two treatment conditions within the leaf L which is determined by the covariate pattern x:

$$\hat{\tau_{b}}\left(x_{i}\right)=\frac{1}{\left|{i:W}_{i}=1,X_{i}\in L\right|}\sum_{i:X_{i}\in L\left(x_{i}\right)}Y_{i}-\frac{1}{\left|{i:W}_{i}=0,X_{i}\in L\right|}\sum_{i:X_{i}\in L\left(x_{i}\right)}Y_{i},$$

where $W_{i}$ indicates treatment assignment (1: active treatment, 0: treatment as usual). A causal forest is built as an ensemble of B such trees and the final estimate is given by averaging the estimates across trees by:

$$\hat{\tau }\left(x_{i}\right)=\frac{1}{B}\sum_{b}^{B}\hat{\tau_{b}}\left(x_{i}\right).$$

The associated uncertainty around the point estimate $\hat{\tau }$ was quantified by constructing 95% confidence intervals using the estimated standard errors: [$\hat{\tau }$ − 1.96 × standard error, $\hat{\tau }$ + 1.96 × standard error]. To provide a holistic view of the distribution and spread of effects across participant characteristics, we constructed figures that displayed $\hat{\tau }$ point estimates for all covariate points in the data (i.e., considering each observed realization of X in the sample) alongside their respective 95% confidence intervals. Observations were organized in ascending order based on their $\hat{\tau }$ values, ranging from the lowest to the highest. Analysis was repeated separately for each type of psychosocial treatment.

To investigate the presence of heterogeneity in treatment effects based on observed covariates, we conducted a descriptive analysis. Specifically, we compared the variance of the Conditional Average Treatment Effect (CATE), calculated from individual treatment effect estimates using a causal forest model, with the total outcome variability, defined as ${\left[\text{SD}\left(Y_{1}\right)+\text{SD}\left(Y_{0}\right)\right]}^{2}$. The total outcome variability was determined by summing the squared standard deviations of outcomes within both the treatment and control groups. This comparison allowed us to evaluate the relative magnitude of treatment effect heterogeneity against the inherent variability in outcomes, providing insights into the presence and extent of HTE.

Additionally, we used the “test_calibration” function from the “grf” package in R. This function provides an omnibus test for detecting systematic variations in the treatment effect, helping us examine whether the treatment's impact differed systematically based on the observed covariates. A coefficient of 1 for the heterogeneity calibration statistic would suggest that the heterogeneity estimates from the forest are well‐calibrated, meaning that the model is effectively capturing variations in treatment effects across different subgroups within the data. The associated *t*‐value measures how much the observed heterogeneity deviates from the expected under the null hypothesis of no heterogeneity. A significantly large positive *t*‐value (> 1.96) indicates statistically significant heterogeneity in treatment effects.

In addition to the omnibus test, we applied the Rank‐Weighted Average Treatment Effect (RATE) methodology using the “rank_average_treatment_effect” function from the “grf” package (Yadlowsky et al. 2021). RATE estimates heterogeneity in treatment effects by prioritizing individuals based on their predicted CATE scores, thereby focusing on subgroups with potentially higher treatment effects. The results are presented in Table S1. This approach provides a complementary perspective to the omnibus test by detecting heterogeneity in targeted subgroups that may not be evident when examining overall treatment effect variability alone.

The omnibus test, as well as the RATE analysis for heterogeneity, however, may not detect subtle heterogeneity in treatment effects. These tests are often designed to identify broad, systematic differences in treatment effects across the entire sample. Heterogeneity could exist in smaller subgroups or be influenced by specific covariate interactions that are not captured by the broad strokes of the omnibus test. Therefore, we employed the CATE best linear projection procedure to approximate the non‐linear relationships captured by the causal forest in a linear framework, which enabled us to examine the contribution of each covariate to the CATE. We fit a linear model to the estimated treatment effects obtained from the causal forest, which enabled us to assess the relationship between various covariates and the estimated individual‐level treatment effects. Essentially, this analysis provided insights into how specific characteristics of individuals, as captured by the covariates, are associated with variations in the effectiveness of the intervention, thereby enhancing the interpretability of the treatment effect heterogeneity uncovered by the causal forest model. A Bonferroni correction was applied to adjust the significance thresholds for the number of covariates used in the model for each intervention.

In SUD trials, early dropout is common, and treatment retention often serves as the primary outcome. Drug use‐related outcomes, such as abstinence, are important but are only observed for participants who remain in the trial, leading to missing outcomes for those who drop out. To account for missing outcomes in our CATE estimation, we conducted sensitivity analyses to account for missing outcomes in our CATE estimation to examine the robustness of our findings. Specifically, these analyses explored three extreme scenarios: one where all missing outcomes were assumed to be non‐abstinent (zero), another where they were considered abstinent (one), and a third, more extreme scenario, where missing outcomes were differentially assumed based on group allocation—treated as abstinent (one) for the treatment group and as non‐abstinent (zero) for the control group. This approach allowed us to assess the impact of varying assumptions about missing data on our estimates of treatment effects (Mayer et al. 2020).

## 3 Results

### 3.1 CATE Estimates

Before presenting estimates of the CATE, we consider the ATE. The motivational incentive intervention displayed a statistically significant positive average treatment effect (ATE 0.15, 95% CI 0.07, 0.24) while the other eight interventions evaluated did not exhibit statistically significant ATEs.

**FIGURE 1:**
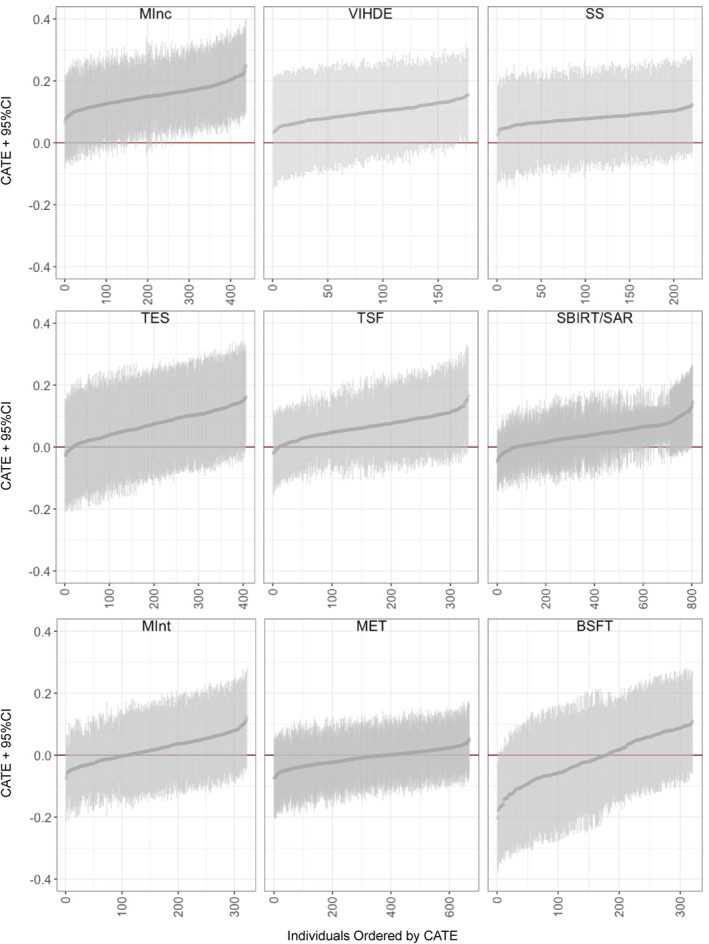
Conditional average treatment effect estimates with 95% confidence intervals.

Figure 1 presents the out‐of‐bag estimate of CATE ($\hat{\tau }$) for each covariate pattern seen in the data for each of the nine psychosocial interventions. Table 2 presents the ATE with its standard error, the variance of the CATE, and the total outcome variability measure (i.e., ${\left[\text{SD}\left(Y_{1}\right)+\text{SD}\left(Y_{0}\right)\right]}^{2}$) for each intervention. Across the nine interventions, the CATE variance consistently showed a smaller range (0.0003–0.0053) compared to the broader total outcome variability (0.387–0.996), reflecting a significant difference in the scale of treatment effect variability and inherent variability within treatment and control groups. Consistently, as shown in Table 3, the heterogeneity calibration statistic generated from the causal forest omnibus test was not significant for any of the interventions, indicating a lack of strong evidence of HTE.

**TABLE 2:** Average and conditional average treatment effect estimates with associated variability measures.

Intervention	ATE	ATE std error	CATE variance	Combined outcome variability^a^
MInc	0.152	0.043	0.0012	0.952
VIHDE	0.102	0.064	0.0007	0.715
SS	0.081	0.064	0.0003	0.996
TES	0.073	0.051	0.0019	0.986
TSF	0.065	0.044	0.0013	0.671
SBIRT/SAR	0.041	0.024	0.0012	0.387
MInt	0.020	0.050	0.0016	0.884
MET	−0.009	0.034	0.0006	0.898
BSFT	−0.012	0.056	0.0053	0.958

Abbreviations: ATE, average treatment effect; BSFT, brief strategic family therapy; CATE, conditional average treatment effect; CV, coefficient of variation; MET, motivational enhancement treatment; MInc, motivational incentive; MInt, motivational interviewing; SAR, screening assessment and referral; SBIRT, screening brief intervention and referral to treatment; SS, seeking safety; TES, therapeutic education system; TSF, twelve‐step facilitation intervention; VIHDE, vigorous intensity high dose exercise.

^a^ This measure was calculated as [SD(*Y*_1_) + SD(*Y*_0_)]^2^, where SD(*Y*_1_) is the standard deviation of outcomes in the treatment group and SD(*Y*_0_) is the standard deviation of outcomes in the control group.

**TABLE 3:** Calibration of predicted treatment effect heterogeneity in causal forest analysis.

Intervention	Heterogeneity calibration statistic	Std error	*t* value	*p* Value
MInc	−1.67	1.29	−1.30	0.90
VIHDE	−7.31	2.11	−3.47	1.00
SS	−23.21	2.39	−9.73	1.00
TES	−3.83	1.09	−3.53	1.00
TSF	−1.67	1.22	−1.37	0.91
SBIRT/SAR	−1.18	0.70	−1.68	0.95
MInt	−0.46	1.23	−0.37	0.64
MET	−5.35	1.28	−4.17	1.00
BSFT	−0.29	0.78	−0.37	0.64

*Note:* Significant calibration statistic suggests that the causal forest model has captured treatment effect heterogeneity.

Abbreviations: BSFT, brief strategic family therapy; MET, motivational enhancement treatment; MInc, motivational incentive; MInt, motivational interviewing; SAR, screening assessment and referral; SBIRT, screening brief intervention and referral to treatment; SS, seeking safety; TES, therapeutic education system; TSF, twelve‐step facilitation intervention; VIHDE, vigorous intensity high dose exercise.

Furthermore, our sensitivity analyses, which accounted for potential variations due to missing outcomes, supported these findings. When assuming three distinct scenarios—all missing outcomes being considered as non‐abstinence, all as abstinence, and the third scenario where missing outcomes were assumed as abstinence in the treatment group and non‐abstinence in the control group (Figures S1–S3)—the resultant CATE projections consistently mirrored our primary analysis.

### 3.2 RATE Analysis

As shown in Table 4, our RATE analysis revealed a statistically significant RATE estimate only for the motivational interviewing intervention (CTN0005), indicating potential treatment effect heterogeneity within this group. Specifically, the RATE estimate for motivational interviewing was 0.13 (95% CI: 0.02, 0.25), suggesting that individuals with higher predicted CATE scores—those whom the causal forest model prioritized as more responsive—may experience greater benefits from this intervention. In contrast, RATE estimates for the other eight interventions were not statistically significant, aligning with the heterogeneity calibration analysis, which did not detect broad patterns of heterogeneity across these interventions.

**TABLE 4:** Results of the rank‐weighted average treatment effect (RATE) analysis for heterogeneity in SUD interventions.

Intervention	RATE estimate	95% confidence interval	*p* Value
MInc	0.00	−0.11, 0.12	0.94
VIHDE	−0.02	0.08, −0.18	0.76
SS	−0.07	−0.25, 0.11	0.47
TES	−0.10	−0.23, 0.03	0.10
TSF	−0.00	−0.13, 0.12	0.96
SBIRT/SAR	−0.03	−0.10, 0.05	0.53
MInt	0.14	0.02, 0.25	0.02
MET	−0.06	−0.14, 0.03	0.21
BSFT	−0.01	−0.15, 0.13	0.89

*Note:* A significant RATE estimate indicates that the causal forest model has successfully identified treatment effect heterogeneity, with certain subgroups exhibiting differential responses to treatment.

Abbreviations: BSFT, brief strategic family therapy; MET, motivational enhancement treatment; MInc, motivational incentive; MInt, motivational interviewing; SAR, screening assessment and referral; SBIRT, screening brief intervention and referral to treatment; SS, seeking safety; TES, therapeutic education system; TSF, twelve‐step facilitation intervention; VIHDE, vigorous intensity high dose exercise.

### 3.3 CATE Best Linear Projection

The best linear projection analysis of the CATE (Table S1) suggests potential associations between CATE and several baseline variables, while holding the other baseline variables fixed. We consider what such associations would mean if the relationship among variables was truly linear, that is, under the assumption of a linear model. For example, the estimated association between the CATE and the ASI employment score is 0.32 (95% CI 0.00, 0.64) for the motivational incentive intervention (combined data of CTN0006 and CTN0007). The interpretation is that for each one‐unit increase in the ASI employment composite score, there is an increase in the CATE by an average of 0.32 units (while holding other baseline variables in the model fixed). In the context of the 12‐step facilitation intervention (CTN0031), the estimated CATE is on average 0.51 units lower (95% CI −0.86,−0.17)—for the group receiving a pension for physical or psychiatric disabilities compared to those not receiving such a pension. Similarly, for the motivational enhancement treatment intervention (combining data from CTN0004, CTN0013, and CTN0021), the estimated CATE is 0.70 units (95% CI −1.22, −0.17) lower for those who selected race category “other” compared to the combined set of categories: “non‐Hispanic White,” “Black,” and “Hispanic.” This suggests the possibility of differential response to the treatment between these groups. Lastly, in the brief strategic family therapy intervention (CTN0014), an estimated association was noted between the CATE and the ASI alcohol score, with a coefficient of −1.84 (95% CI −3.41, −0.27), suggesting a possible decrease in the CATE by an average of 1.84 units for each one‐unit increase in the ASI alcohol score. After applying a Bonferroni correction for multiple comparisons, however, none of these observed associations remained statistically significant. Also, the above interpretations all rely on the linearity assumption given above.

## 4 Discussion

This study was the first application of a causal forest approach to the SUD field for the purpose of examining HTE within existing SUD treatment RCTs. Using causal forests, we estimated the CATE on drug abstinence across multiple datasets. To examine the presence of HTE, we compared the CATE variance against the total outcome variability, a measure of inherent variability within treatment and control groups and conducted an omnibus test. While the CATE variance was consistently lower than the combined outcome variability across nine interventions, suggesting a lack of strong evidence for broad HTE, the RATE analysis indicated potential subgroup‐specific heterogeneity within the motivational interviewing intervention. This result suggests that certain subgroups, particularly those with higher predicted CATE scores, may experience differential treatment effects. These findings underscore the utility of combining RATE with calibration tests to capture subtle, subgroup‐specific heterogeneity that may not be detectable through standard variance‐based measures alone.

This study does have limitations. Given the limited sample size for each intervention, with randomized trials generally powered to detect overall effects rather than CATEs, our statistical power was constrained, potentially limiting the detectability of subtle heterogeneities. Given that we did not find strong evidence of the existence of such predictors among the variables that we considered, future studies would need to have substantially larger sample sizes in order to identify such predictors (if they exist) or have different sets of variables.

In our analysis, all samples were drawn from a single clinical network, which raises concerns regarding the generalizability of our findings. It is plausible that the patterns of HTE might differ in other settings or populations or non‐abstinence‐based outcomes such as reduced drug use. Another limitation was the restricted set of baseline covariates available for our analysis. Critical variables such as comorbid mental disorders (e.g., depression, anxiety), personality traits (e.g., neuroticism, conscientiousness), and cognitive factors (e.g., executive functioning, working memory), which could significantly influence treatment outcomes, were not available for this analysis. Additionally, factors such as patients' willingness or motivation for treatment, which are likely to impact the effectiveness of psychosocial interventions, were also missing. Lastly, while the analysis adjusts for multiple comparisons across covariates within each intervention, it does not extend this adjustment across the nine interventions included. Thus, interpretations of findings should be approached with caution when comparing across different interventions. Therefore, the RATE analysis for the motivational interviewing intervention should be interpreted cautiously, as this was an exploratory analysis involving multiple hypotheses (nine separate RATE analyses). This finding could represent a false positive, and replication would be necessary (i.e., an additional study) to confirm this result.

Our results also highlight the importance of diverse, larger‐scale studies that consider a broader spectrum of covariates and have higher statistical power to detect effect heterogeneity. As the field continues to evolve, studies like ours help guide the way for future studies to potentially uncover a more nuanced understanding of treatment effects for substance use disorders.

## Author Contributions

**Ryoko Susukida:** conceptualization, investigation, funding acquisition, writing–original draft, methodology, software, data curation, supervision, formal analysis, visualization, writing–review & editing. **Masoumeh Amin‐Esmaeili:** conceptualization, methodology, data curation, software, investigation, writing–review & editing. **Elena Badillo‐Goicoechea:** methodology, software, investigation, visualization, writing–review & editing. **Trang Q. Nguyen:** conceptualization, methodology, software, visualization, writing–review & editing, funding acquisition. **Elizabeth A. Stuart:** writing–review & editing, conceptualization, funding acquisition, supervision, methodology. **Michael Rosenblum:** conceptualization, funding acquisition, writing–review & editing, methodology. **Kelly E. Dunn:** conceptualization, funding acquisition, writing–review & editing, resources. **Ramin Mojtabai:** conceptualization, funding acquisition, writing–review & editing, supervision, resources, investigation.

## Conflicts of Interest

Dr. Susukida reported grants from the NIDA and the American Foundation for Suicide Prevention during the conduct of the study. Dr. Mojtabai has received royalties and consulting fees from UpToDate, Medscape and MindMed. Dr. Kelly Dunn has consulted with Cessation Therapeutics, DemeRx, and Mind Med in the past 3 years and received funding from NIDA and Cure Addiction Now.

## Supporting information

Figures S1–S3

Table S1

## Data Availability

The data that support the findings of this study are openly available in the National Institute on Drug Abuse Data Share Website at https://datashare.nida.nih.gov, reference number NIDA‐CTN‐0004, NIDA‐CTN‐0005, NIDA‐CTN‐0006, NIDA‐CTN‐0007, NIDA‐CTN‐0013, NIDA‐CTN‐0014, NIDA‐CTN‐0015, NIDA‐CTN‐0021, NIDA‐CTN‐0031, NIDA‐CTN‐0044, and NIDA‐CTN‐0047.
