# Supplementary material for: Application of Causal Forest Model to Examine Treatment Effect Heterogeneity in Substance Use Disorder Psychosocial Treatments
Source: Int J Methods Psychiatr Res. 2024 Dec 27;34(1):e70011. doi: 10.1002/mpr.70011 (PMC11675088; doi:10.1002/mpr.70011)

Supplementary Figure 1 Sensitivity analysis of CATE with an assumption of all missing outcomes being zero

CATE + 95%CI


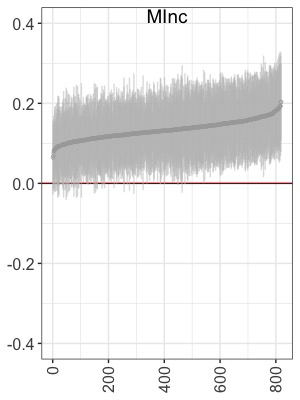

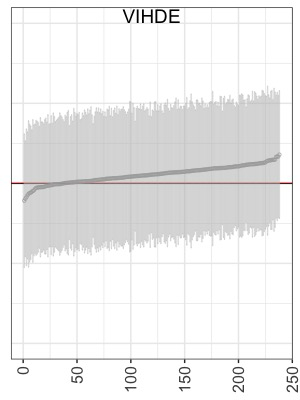

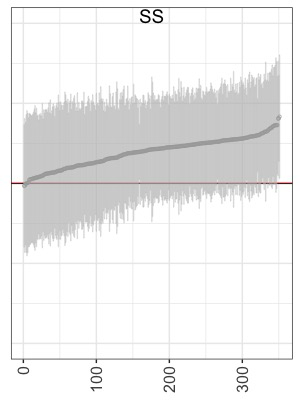

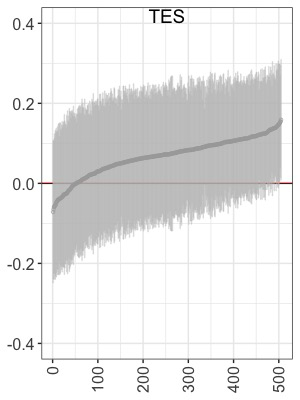

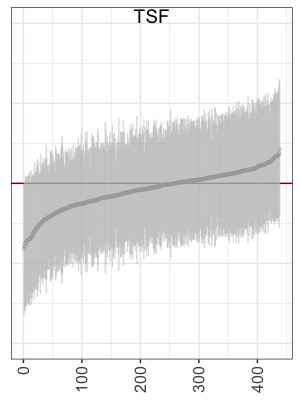


CATE + 95%CI


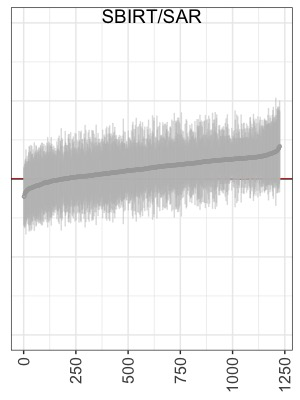

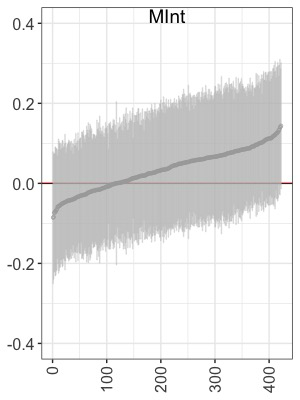


CATE + 95%CI


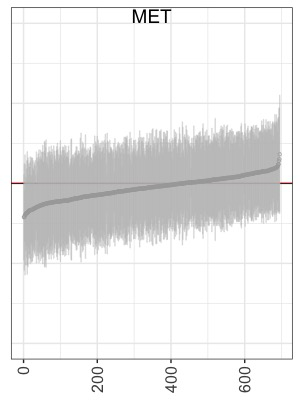

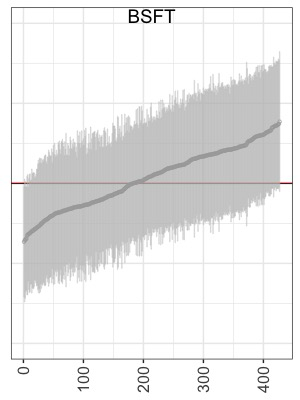


Individuals Ordered by CATE

Supplementary Figure 2 Sensitivity analysis of CATE with an assumption of all missing outcomes being one


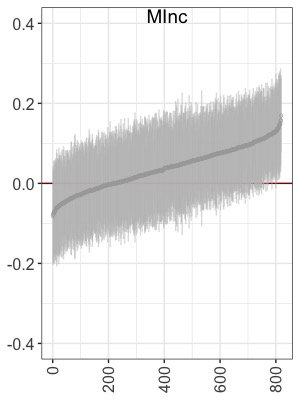


CATE + 95%CI


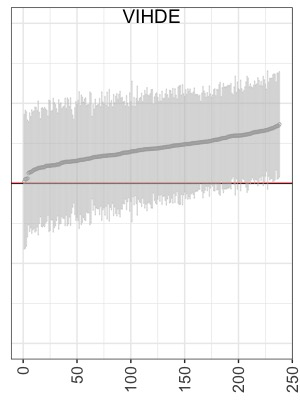

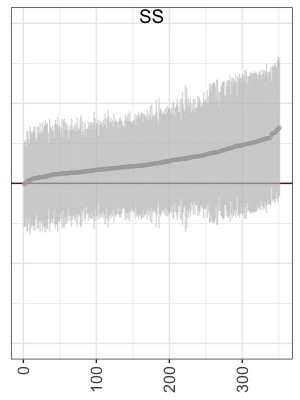

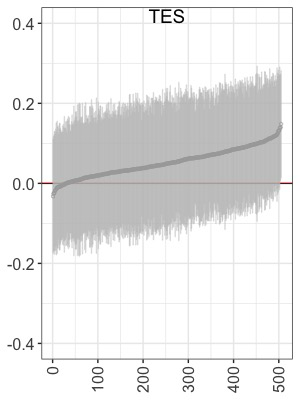


CATE + 95%CI


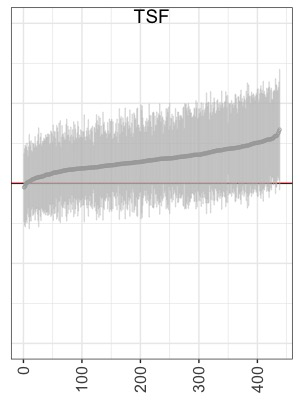

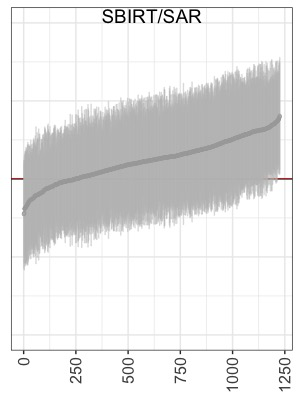

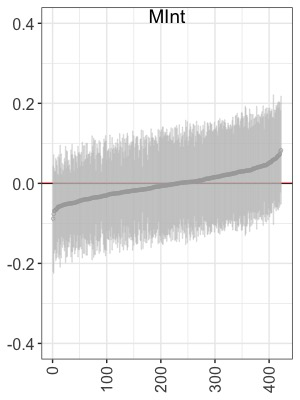

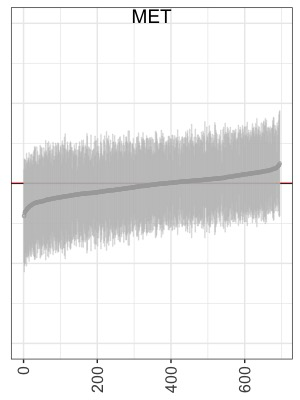

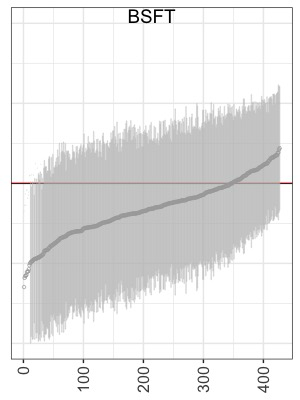


CATE + 95%CI

Individuals Ordered by CATE

CATE + 95%CI

Supplementary Figure 3 Sensitivity analysis of CATE with differential assumptions of missing outcomes: one in treatment group and zero in control group

CATE + 95%CI


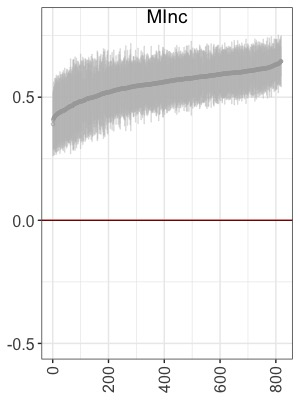

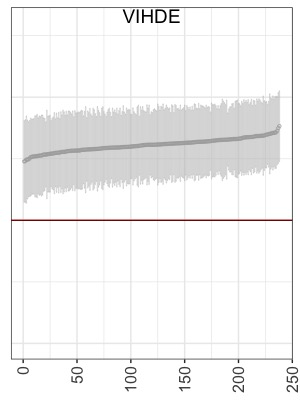

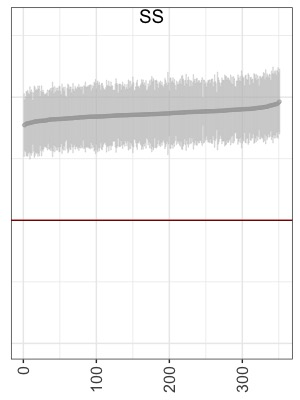

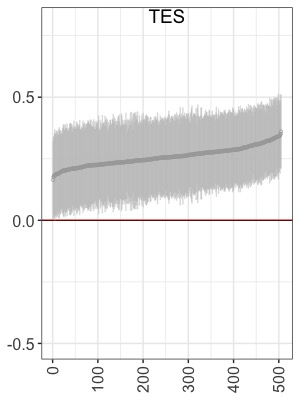

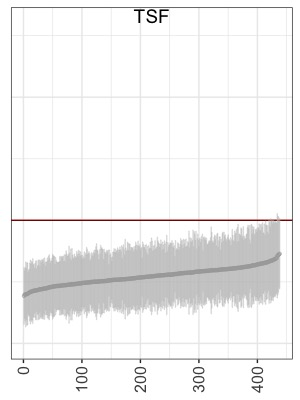

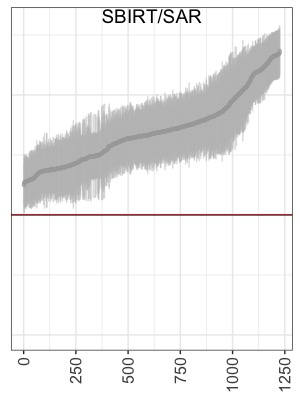


CATE + 95%CI

Individuals Ordered by CATE

CATE + 95%CI


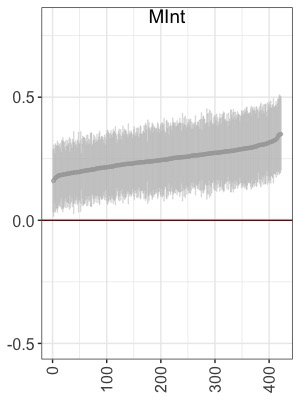

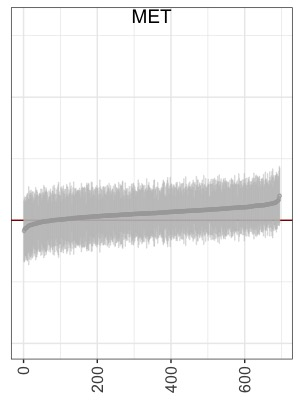

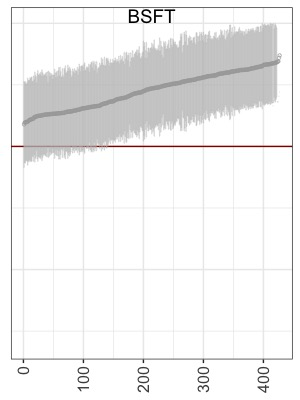

Supplement: Supplementary file 1 — Figures S1–S3 [file MPR-34-e70011-s002.docx]
