# Supplementary material for: Application of Causal Forest Model to Examine Treatment Effect Heterogeneity in Substance Use Disorder Psychosocial Treatments
Source: Int J Methods Psychiatr Res. 2024 Dec 27;34(1):e70011. doi: 10.1002/mpr.70011 (PMC11675088; doi:10.1002/mpr.70011)
Supplement: Supplementary file 2 — Table S1 [file MPR-34-e70011-s001.docx]

| Supplemental Table 1. Best linear projection estimates of the associations between the CATE and baseline covariates | | | | | | | | | |
| --- | --- | --- | --- | --- | --- | --- | --- | --- | --- |
|  | MInc | VIHDE | SS | TES | TSF | SBIRT/SAR | MInt | MET | BSFT |
|  | Coeff.  95%CI | Coeff.  95%CI | Coeff.  95%CI | Coeff.  95%CI | Coeff.  95%CI | Coeff.  95%CI | Coeff.  95%CI | Coeff.  95%CI | Coeff.  95%CI |
| Age | 0.00  (-0.01, 0.01) | -0.01  (-0.02, 0.01) | 0.01  (-0.01, 0.02) | 0.00  (-0.01, 0.01) | 0.00  (-0.01, 0.01) | 0.00  (0.00, 0.00) | 0.00  (-0.01, 0.01) | 0.00  (-0.01, 0.01) | 0.07  (-0.02, 0.16) |
| Female | 0.17  (-0.04, 0.38) | -0.29  (-0.63, 0.05) | --^1^ | -0.14  (-0.35, 0.08) | 0.13  (-0.09, 0.35) | 0.07  (-0.03, 0.17) | 0.10  (-0.15, 0.35) | -0.04  (-0.24, 0.16) | 0.16  (-0.13, 0.46) |
| Race, Black | -0.09  (-0.35, 0.17) | 0.05  (-0.33, 0.43) | 0.15  (-0.22, 0.51) | -0.06  (-0.32, 0.21) | 0.09  (-0.14, 0.32) | -0.07  (-0.21, 0.07) | -0.31  (-0.68, 0.06) | -0.16  (-0.38, 0.07) | -0.19  (-0.52, 0.15) |
| Race, Other | -0.06  (-0.44, 0.33) | -0.09  (-0.79, 0.60) | 0.20  (-0.27, 0.68) | -0.02  (-0.33, 0.28) | 0.19  (-0.15, 0.52) | -0.01  (-0.23, 0.21) | -0.06  (-0.38, 0.25) | -0.70  (-1.22, -0.17) | 0.09  (-0.41, 0.60) |
| Hispanic | 0.19  (-0.10, 0.48) | 0.17  (-0.27, 0.61) | -0.08  (-0.73, 0.56) | 0.10  (-0.23, 0.43) | -0.24  (-0.57, 0.10) | -0.02  (-0.17, 0.13) | -0.21  (-0.93, 0.50) | -0.15  (-0.36, 0.06) | -0.02  (-0.52, 0.15) |
| ≥12 years education | 0.08  (-0.11, 0.27) | 0.26  (-0.22, 0.74) | -0.04  (-0.38, 0.30) | -0.10  (-0.34, 0.14) | -0.14  (-0.36, 0.08) | 0.04  (-0.05, 0.13) | 0.07  (-0.20, 0.35) | 0.04  (-0.11, 0.20) | --^2^ |
| Fulltime job | 0.00  (-0.21, 0.20) | 0.13  (-0.17, 0.44) | 0.11  (-0.19, 0.42) | -0.08  (-0.29, 0.14) | 0.08  (-0.12, 0.28) | 0.01  (-0.13, 0.16) | -0.20  (-0.44, 0.03) | 0.01  (-0.15, 0.17) | --^2^ |
| Alone or unstable  living arrangement | -0.04  (-0.26, 0.18) | -0.18  (-0.52, 0.16) | 0.25  (-0.10, 0.59) | -0.19  (-0.45, 0.07) | 0.12  (-0.11, 0.35) | --^2^ | 0.00  (-0.29, 0.30) | -0.03  (-0.24, 0.19) | --^2^ |
| Religious preference | -0.09  (-0.33, 0.14) | 0.02  (-0.32, 0.36) | 0.00  (-0.32, 0.32) | --^2^ | -0.05  (-0.26, 0.16) | --^2^ | -0.08  (-0.29, 0.14) | 0.04  (-0.13, 0.20) | --^2^ |
| Prior alcohol/drug  treatment | 0.19  (-0.12, 0.51) | 0.16  (-0.62, 0.93) | 0.19  (-0.41, 0.78) | --^2^ | 0.06  (-0.45, 0.57) | --^2^ | 0.12  (-0.10, 0.34) | 0.06  (-0.10, 0.22) | -0.05  (-0.42, 0.33) |
| Pension for physical  and/or psychiatric disabilities | 0.22  (-0.01, 0.46) | -0.30  (-0.90, 0.30) | -0.09  (-0.65, 0.47) | --^2^ | -0.51  (-0.86, -0.17) | --^2^ | 0.12  (-0.32, 0.57) | -0.08  (-0.38, 0.21) | --^2^ |
| Physical and/or sexual  abuse history | -0.15  (-0.37, 0.07) | -0.09  (-0.47, 0.28) | 0.21  (-0.60, 1.02) | --^2^ | 0.03  (-0.19, 0.26) | --^2^ | 0.13  (-0.11, 0.37) | 0.06  (-0.13, 0.25) | --^2^ |
| Injection drug use | -0.04  (-0.24, 0.16) | -0.17  (-0.53, 0.19) | -0.28  (-0.72, 0.16) | -0.36  (-0.81, 0.09) | -0.02  (-0.22, 0.17) | -0.03  (-0.17, 0.12) | 0.09  (-0.34, 0.52) | -0.20  (-0.54, 0.15) | 0.11  (-0.63, 0.86) |
| ASI - Psychiatric | 0.09  (-0.35, 0.52) | 0.56  (-0.18, 1.30) | 0.16  (-0.61, 0.92) | --^2^ | 0.38  (-0.04, 0.79) | --^2^ | 0.25  (-0.29, 0.78) | -0.17  (-0.55, 0.21) | --^2^ |
| ASI - Medical | -0.01  (-0.27, 0.25) | 0.23  (-0.36, 0.81) | -0.18  (-0.66, 0.31) | --^2^ | 0.14  (-0.13, 0.41) | --^2^ | 0.08  (-0.27, 0.43) | 0.05  (-0.23, 0.32) | --^2^ |
| ASI - Employment | 0.32  (0.00, 0.64) | 0.11  (-0.43, 0.64) | -0.37  (-0.90, 0.15) | --^2^ | 0.10  (-0.25, 0.45) | --^2^ | -0.17  (-0.55, 0.20) | 0.11  (-0.14, 0.37) | --^2^ |
| ASI - Alcohol | -0.09  (-0.69, 0.51) | -0.36  (-1.02, 0.31) | -0.27  (-0.91, 0.36) | --^2^ | -0.06  (-0.56, 0.44) | --^2^ | -0.15  (-0.61, 0.30) | 0.18  (-0.17, 0.52) | -1.84**  (-3.41, -0.27) |
| ASI - Drug | -0.40  (-1.11, 0.32) | 0.18  (-1.69, 2.05) | 0.86  (-0.36, 2.09) | --^2^ | 0.55  (-0.53, 1.63) | --^2^ | -0.12  (-1.20, 0.97) | -0.69  (-1.45, 0.06) | 2.69  (-2.01, 7.40) |
| ASI - Legal | -0.04  (-0.55, 0.47) | 0.19  (-0.62, 1.01) | 0.54  (-0.18, 1.25) | --^2^ | -0.13  (-0.63, 0.37) | --^2^ | -0.01  (-0.52, 0.50) | 0.00  (-0.40, 0.40) | --^2^ |
| ASI - Family/Social | -0.15  (-0.57, 0.28) | 0.17  (-0.47, 0.81) | -0.25  (-0.94, 0.44) | --^2^ | -0.03  (-0.40, 0.34) | --^2^ | -0.17  (-0.72, 0.38) | 0.02  (-0.35, 0.39) | --^2^ |
| MET: Motivational Enhancement Treatment; TAU: Treatment as Usual; MInc: Motivational Incentive; SBIRT: Screening Brief Intervention and Referral to Treatment; SAR: Screening Assessment and Referral; MSO: Minimal Screening Only; TES: Therapeutic Education System; BSFT: Brief Strategic Family Therapy; TSF: Twelve-Step Facilitation intervention; MInt: Motivational Interviewing; SS: Seeking Safety; HE: Health Education; VIHDE: Vigorous Intensity High Dose Exercise. 1: The trial of SS included only female participants. 2: Variables were not available. | | | | | | | | | |
